# The Role of Melanoma Cell-Stroma Interaction in Cell Motility, Invasion, and Metastasis

**DOI:** 10.3389/fmed.2018.00307

**Published:** 2018-11-06

**Authors:** Robert J. Ju, Samantha J. Stehbens, Nikolas K. Haass

**Affiliations:** ^1^The University of Queensland, The University of Queensland Diamantina Institute, Translational Research Institute, Brisbane, QLD, Australia; ^2^Discipline of Dermatology, University of Sydney, Sydney, NSW, Australia

**Keywords:** melanoma, motility, invasion, metastasis, microenviroment, cytokines, chemokines, migrastatics

## Abstract

The importance of studying cancer cell invasion is highlighted by the fact that 90% of all cancer-related mortalities are due to metastatic disease. Melanoma metastasis is driven fundamentally by aberrant cell motility within three-dimensional or confined environments. Within this realm of cell motility, cytokines, growth factors, and their receptors are crucial for engaging signaling pathways, which both mediate crosstalk between cancer, stromal, and immune cells in addition to interactions with the surrounding microenvironment. Recently, the study of the mechanical biology of tumor cells, stromal cells and the mechanics of the microenvironment have emerged as important themes in driving invasion and metastasis. While current anti-melanoma therapies target either the MAPK signaling pathway or immune checkpoints, there are no drugs available that specifically inhibit motility and thus invasion and dissemination of melanoma cells during metastasis. One of the reasons for the lack of so-called “migrastatics” is that, despite decades of research, the precise biology of metastatic disease is still not fully understood. Metastatic disease has been traditionally lumped into a single classification, however what is now emergent is that the biology of melanoma metastasis is highly diverse, heterogeneous and exceedingly dynamic—suggesting that not all cases are created equal. The following mini-review discusses melanoma heterogeneity in the context of the emergent theme of mechanobiology and how it influences the tumor-stroma crosstalk during metastasis. Thus, highlighting future therapeutic options for migrastatics and mechanomedicines in the prevention and treatment of metastatic melanoma.

## Introduction

**Figure 1:**
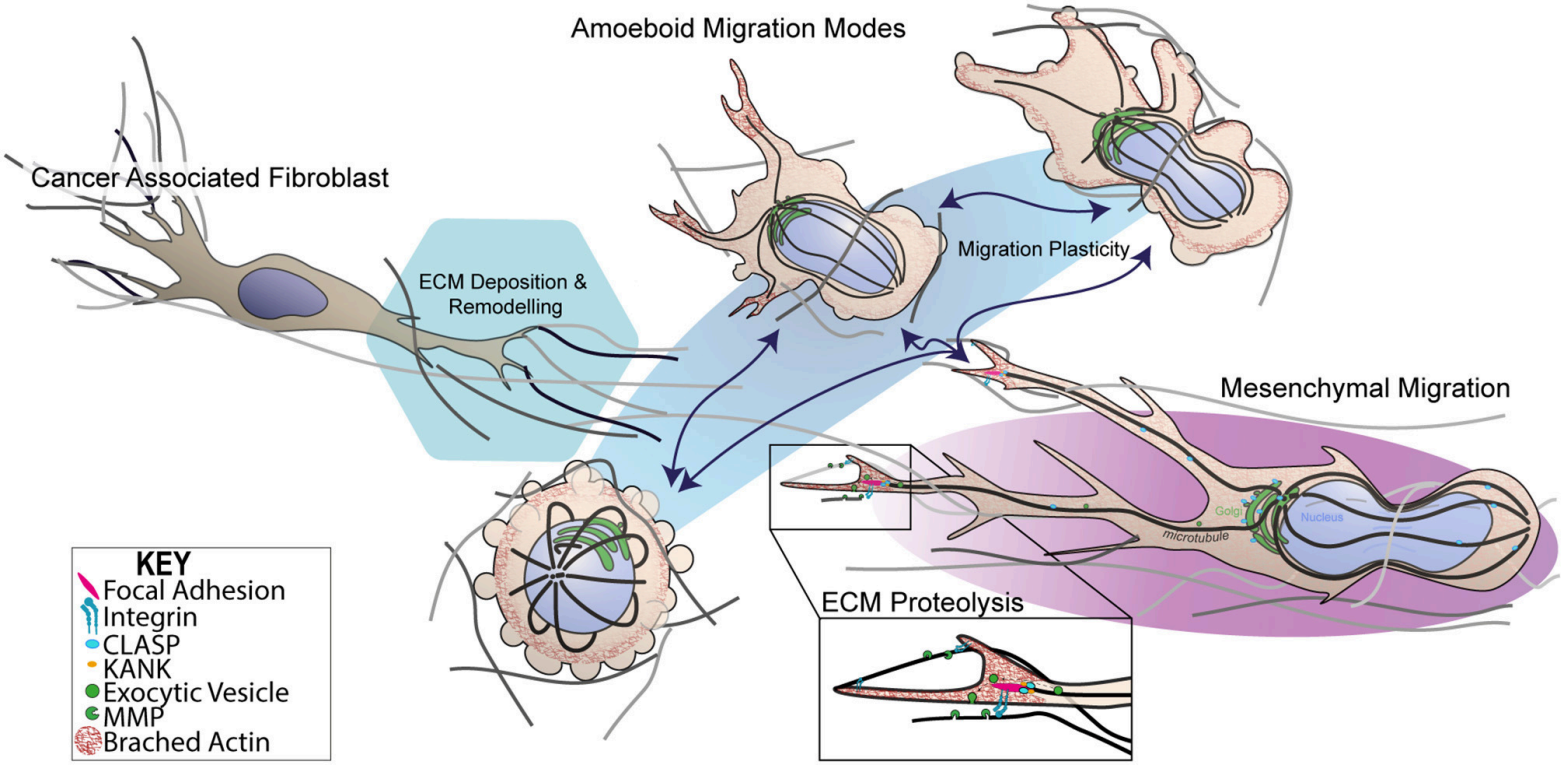
Dynamic switching of 3D migration modes in metastatic melanoma. Schematic representation of 3D mesenchymal and amoeboidal migration mode dynamic switching. Mesenchymal migration relies on RAC signaling to establish front-rear 3D polarity in order to generate a predominant pseudopodia, producing the characteristic spindle like morphology. Mesenchymal migration relies on MMP-dependent ECM degradation and integrin-dependent cell-matrix attachment protein complexes known as focal adhesions (FAs). FAs are thought to be hot spots for exocytic trafficking of MMPs, mediated by cortical microtubule stabilization complexes containing microtubule associated proteins, CLASPs, and FA adaptor proteins, KANKs. In contrast, amoeboid migration subsumes several migration modes from blebbing, chimneying to actin gliding modes. Importantly, amoeboidal migration requires little to no integrin activity and or MMP-mediated matrix degradation. Transformed cancer-associated fibroblasts (CAFs) are known to facilitate ECM changes through deposition of fibronectin that crosslinks collagen I fibers. CAF-dependent contractility further in reorganization of ECM influenceing melanoma migration and survival mechanosensory proteins.

**Figure 2:**
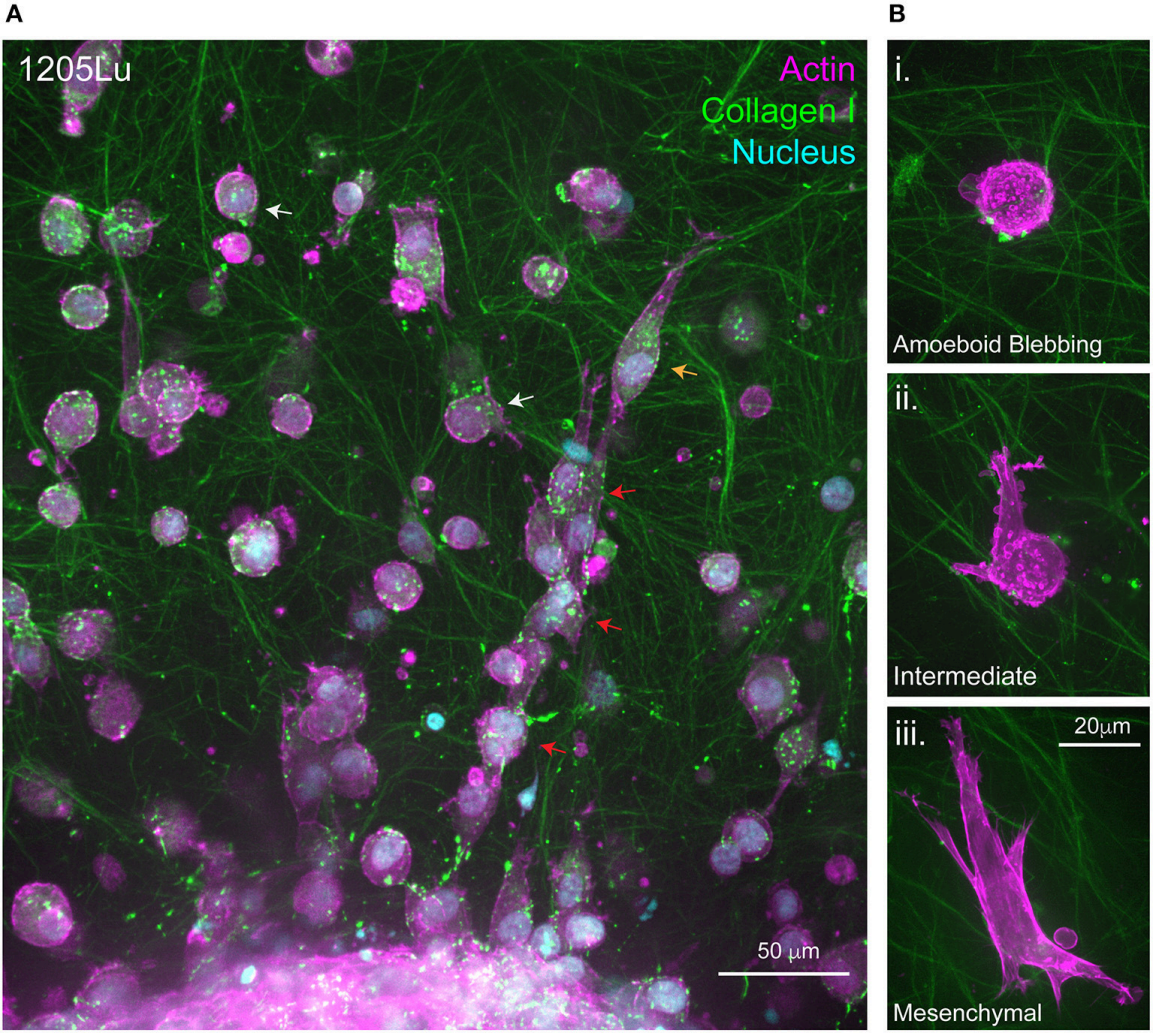
Migration at the 3D Melanoma-collagen interface. **(A)** Melanoma spheroids (1205Lu cells) embedded in a porous fibrillar collagen I hydrogel show heterogeneous 3D migration phenotypes at the spheroid-matrix interface. Within collagen I hydrogels, melanoma cells exhibit multi-cellular streaming (***red arrows***), single-cell rounded migration (***white arrows***) and polarized mesenchymal migration (***orange arrow***). **(B)** Representative high resolution spinning disc confocal images of single melanoma cells expressing fluorescently labeled filamentous actin (mScarlet-i-Lifeact) demonstrating several 3D migration phenotypes within the same collagen I hydrogel **i**. Amoeboid blebbing migration **ii**. Intermediate blebbing-pseudopodia phenotype and **iii**. Mesenchymal migration.

In recent years, studies elucidating the biology of metastatic melanoma have revealed highly complex, yet dynamic processes, with equal parts unpredictability. The dynamic behavior of metastatic melanoma is highlighted by the multiple migration modalities employed to navigate the topography of a fluctuating microenvironment (Figures 1, 2). The ability of melanoma to both transition through multiple environments and integrate stromal signals with invasive and proliferative behaviors influences the response of metastatic melanoma to therapies. Advanced stage metastatic disease is particularly important as it is currently incurable by conventional targeted therapies and patient survival does not benefit from surgical resection alone (1). Furthermore, paradigm shifting “immuno-therapies” whilst effective in a subset of patients, are not effective in all (2–5). Thus, many patients diagnosed with metastatic melanoma are left with little to no treatment options, leaving survival prognosis bleak (1, 6–8). This underscores the importance of both studying and understanding the biology of melanoma, with the aim of using this knowledge to produce novel targeted therapies, or “migrastatics” (9), that specifically target both the cancer and the microenvironment to prevent metastatic spread.

## Metastatic melanoma

Melanoma metastasis is governed by the fundamental process of cell motility, whereby aberrantly transformed cancer cells hi-jack normal cellular processes used in homeostasis and development (10–12). Broadly, tumor cells that have subsumed a pro-invasive phenotype achieve metastatic dissemination through processes of intra- and extravasation to arrest at anatomically distant sites where they regain proliferative programming (13). Although we have progressively elucidated our understanding of metastatic behaviors, particularly those shared across multiple cancer types, the exact molecular mechanisms remain elusive and are of high clinical relevance (14–17). Metastatic melanoma spreads in an unpredictable fashion, often through parallel routes of widespread dissemination to multiple organs (18). Melanoma has been shown to engage in conventional intravasation and subsequent vascular and lymphovascular metastatsis (19) and also unconventional intravasation-independent migration known as “pericytic mimicry” (20, 21), where tumor cells migrate along the exterior of blood vessels. There have even been reports of melanoma cells exhibiting exhibiting “vasculogenic mimicry,” whereby they are proposed to form *de novo* vascular networks to promote tumor perfusion (22). Interestingly, parallels exist between the highly invasive nature of metastatic melanoma and their neural crest/melanoblast precursors, with the two sharing similar pro-migratory behavior attributes resulting in multiple studies suggesting that melanoma reactivates neural crest migration programs to drive plasticity and invasiveness in melanoma (12, 18, 23, 24).

## Involvement of cytokines and chemokines in melanoma metastasis

Despite dissemination to most tissue types, melanoma exhibits metastatic tropism, preferentially metastasizing to the brain, lung, liver, small bowel or skin (25). Although the specific tumor-tissue tropism mechanisms are still unclear; chemokine receptors appear to play a role in tumor-tissue “homing” (26, 27). Recent studies show that cytokines and chemokines are integral to immune detection of melanoma cells by differentially regulating the behavior of monocytes, macrophages and natural killer cells (NK cells) (27, 28). Normally, these immune cells function to detect and kill pre-metastatic tumor cells. This process is mediated by the type 2 tumor suppressor protein, pigment epithelium-derived factor (PEDF), whereby PEDF-positive tumor-derived exosomes circulate the vasculature and mount immune responses. This results in, (1) macrophage differentiation and tumor cell detection through the modulation of the IL-10/12 axis, as well as (2) the recruitment of CX3CR1-expressing patrolling monocytes, which function to clear micro-particles and cellular debris from the microvasculature. Additionally, the recruitment and activation of NK cells has been shown to play an auxiliary role in tumor cell killing. The activation of these three arms results in immune detection of pre-metastatic melanoma cells ensuing in tumor death and clearance (27). However, PEDF expression in tumor cells and circulating exosomes is lost during metastatic melanoma transformation, and thus metastatic cells go undetected, allowing cellular debris and micro-particles to create pre-metastatic niches at distant microenvironments (27, 29–33). This process involves transforming and modulating local inflammatory immune cells, stromal cells and extracellular matrix (ECM) through the secretion of homing factors, inflammatory cytokines, and chemokines (34–36). Reciprocally, melanoma secreted cytokines and progressive increases in chemokine receptor expression during progression act to drive angiogenesis and metastasis to certain organs, respectively (37–39). Specifically, studies have shown that the ectopic expression of the chemokine receptor CCR7 in murine melanoma cells increases tumor-lymph node and -brain tissue homing *in vivo* (40), whilst CXCR4 promotes melanoma-lung tropism (41). However, melanoma tissue tropism is likely to be more complex as studies using human melanoma xenografts only partially recapitulate this phenomenon (42). Irrespectively, these findings demonstrate that chemokines play a role in the “tissue-homing,” supporting Paget's 1889 “Seed and Soil” hypothesis that postulated tumor metastasis to particular anatomical sites was driven by cellular mechanism, and not at random (43, 44).

## The role of cell motility and microenvironment mechanics in melanoma invasion

The phenotype-switching model of melanoma heterogeneity (45–47) highlights the importance of understanding the influence of the microenvironment on invasive behavior, notably, how do cells move in 3D? 3D cell motility is a complex biophysical process, which occurs through dynamic interplay between cytoskeletal remodeling, plasma membrane deformation, acto-myosin contractility, and cell-matrix adhesion. The functional organization of these molecular components is highly adaptive, mechanically responsive and varies between cell and tissue types (48–50). The theme of mechanoreciprocity encompasses the rapidly growing knowledge that the cell-ECM interaction is in fact a bi-directional relationship resulting in a biophysical reciprocity whereby cancer cells switch between a growing repertoire of migration modalities to adapt to the geometry, topography, elasticity and chemical composition their surrounding microenvironment (Figure 1). The bi-direcitonal nature of mechanoreciprocity arrives from our understanding of the multi-component viscoelastic units (i.e., cells and the ECM), subject to reciprocal mechanical and chemical interactions that catalyze, assist or restrict cell migration, in context dependent manners (51). This relationship acts to facilitate changes in cell shape, position, the decision between single-cell and collective migration, and structural modification to tissue and the microenvironment through the deposition of matrix (52). The question of how melanoma cells interpret particular environmental cues and integrate a response hierarchy to each stimulus remains an exciting area with the capacity to influence therapies (9, 53).

## Cytoskeletal driving forces of motility

Cell motility is driven by a symphony of cytoskeletal components, which act in fine spatial-temporal orchestration to facilitate movement. The cytoskeleton encompasses actin microfilaments, microtubules, intermediate filaments, and septins (48, 54–58). The spotlight for motility has long been held by the actin cytoskeleton, with many researchers proposing that targeting the actin cytoskeletal network during invasion, through rate limiting mechanisms, may abrogate cancer metastasis and invasion (9, 59–62). A substantial amount of our cell motility knowledge has been uncovered by studying cultured cells on stiff 2D substrates such as plastic and glass (63, 64). However, the greatest limitation that 2D migration biology imposes is that it very poorly recapitulates the biomechanical architecture within tissues and organs in mammalian tissue (63, 65, 66) which varies dramatically in bio-mechanical composition and thus impacts on mechanosensing (67). Similar to other 3D models of cancer, our studies, have demonstrated that 3D models more accurately mirror *in vivo* biology and tumor drug response when studying melanoma (68–71).

## 3D migration plasticity

Similarly to 2D, cell shape changes observed in 3D migration are classically attributed to remodeling of the actin cytoskeleton and spatio-temporal regulation of RHO/RAC GTPase signaling that govern cytoskeletal dynamics (72). Traditionally, cancer cells predominantly exploit two modes of 3D or confined migration, categorized as either amoeboidal or mesenchymal migration (Figure 1) (73). More complex models have identified that cancer cells can also behave semi-collectively to exhibit multicellular streaming whereby cells invade in a chain-like fashion following a primary leader cell (Figure 2) (52). It is the ability of cells when subjected to different 3D ECM topographies to dynamically adapt to undergo the most efficient mode of migration (51) (often switching between migration mode sub-types), which highlights the role of mechanoreciprocity in facilitating dynamic plasticity during cancer invasion

One crucial feature that affects the cell determination of migration mode is the degree of ECM confinement (74). Increased confinement imposes a mechanical cell-deformation challenge, which allows cells to traverse through narrow spaces. Several landmark studies have demonstrated that the degree to which cancer cells are able to squeeze to facilitate migration is rate-limited by the degree of nuclear compression or distortion, defined as the “nuclear limit of migration,” which is equal to approximately 10% of the nuclear cross-sectional area (75, 76). If nuclear constriction falls beyond this limit, cells switch to migration modalities that upregulate ECM proteolysis, seen in the process of amoeboid-to-mesenchymal switching (50, 75, 77) or alternatively undergo nuclear envelope rupture which may affect cell viability (78–80).

## Adhesion-independent amoeboidal migration

Blebbing behavior for motility purposes was overlooked until a landmark study identified melanoma cells lacking the actin crosslinking protein, Filamin A, exhibited non-apoptotic blebbing which correlated with motility (81). The classification of amoeboid migration now subsumes a heterogeneous spectrum of migration modes ranging from blebbing, “chimneying” and actin-polymerisation gliding-based modes (Figure 1) (82). Amoeboid-based motility is rapid as it entails little to no integrin activity and does not require proteolytic degradation, opening of junctions, penetration of basement membranes, or breaching endothelial vasculature (50, 83). Instead, surrounding surfaces immobilize cells with the transmission of traction forces alone being sufficient for cells movement. The mechanical drivers of amoeboid migration are attributed to the RHOA-ROCK-myosin II signaling axis which governs acto-myosin contractility to generate membrane blebs which are both reinforced by ROCK-dependent JAK-STAT3 signaling and maintained by IL6-STAT3 (84, 85). Myosin II contractile activity drives membrane blebbing by localized rises in hydrostatic pressure resulting in the rupture of cortical actin networks (86) or localized detachment of plasma membrane from the cortical actin cytoskeleton (87). Both mechanisms that generate hydrostatic pressure gradients are able to produce spherical membrane blebbing as a result of cytosolic content flow that protrudes the plasma membrane. The subsequent membrane bleb retraction occurs once pressure is equilibrated (88). Acto-myosin blebbing activity functions in tandem with RHO-RAC inhibitory crosstalk, which generates tractional force through the inter-digitation of blebs or actin based protrusions into gaps within the matrix environment (89). The cytokine TGFβ has also been shown to promote amoeboid migration and invasion in melanoma via a SMAD/CITED-dependent transcriptional activation of key genes involved in sustained actomyosin contractility (90).

## Protease-dependent mesenchymal migration

Mesenchymal or proteolytic-dependent modes of migration occur when cells are subjected to highly confined microenvironments, that are not permissible to squeezing-based migration modes, requiring cells to remodel the matrix to facilitate invasion (50). Cells engaged in mesenchymal migration exhibit polarized spindle-like morphologies, which are highly dependent on integrin-based ECM adhesion and proteolysis of ECM (Figure 1) (91). The characteristic polarized phenotype of mesenchymal migration and also the formation of a predominant pseudopodium, analogous to a lamellipodium in 2D, arises from RAC signaling which results in a slow, but highly directional 3D migration (Figure 2iii) (92–94). Interestingly, the RAC1 GTPase activating mutation (P29S) is the third most frequently occurring mutation in sun-exposed melanoma (~9%). RAC1 plays significant roles in lamella actin dynamics, acting upstream of key actin regulatory proteins including Arp2/3 and Cofilin to drive membrane protrusion in 2D. RAC1^P29S^ drives resistance to targeted therapy (84, 85) via mechanosensory-dendritic actin polymerization in low compliance environments which drives proliferation via a MAPK-independent pathway (95). RAC1 also affects 3D cell motility (86) and inhibits RHO-ROCK signaling, further highlighting that the presence and high frequency of RAC1^P29S^ in melanoma cases may have implications on both melanoma metastasis and survival in these patients.

The pseudopodial extensions are created by polarized actin polymerisation which results in the breaking of symmetry and formation of a dominant protrusion. Pseudopodia a facilitates directional 3D migration whilst also allowing cells to mechanosense their microenvironments. Pseudopodial extensions are stabilized by interactions with the matrix through integrin transmembrane receptor interactions, which scaffold into multiprotein complexes known as focal adhesions (Figures 1, 2iii.). These mechanosensory complexes determine changes in substrate composition and stiffness primarily through the force transduction proteins talin and vinculin which scaffold onto the acto-myosin cytoskeleton (Figure 1) (96). Force-dependent conformational changes in talin act to coordinate recruitment of key focal adhesion signaling and scaffolding proteins [i.e., KANKS (97)] by exposing and disrupting binding-sites in a tension-dependent manner (98). Additionally, focal adhesions serve as ECM anchor points, enabling actin-dependent traction force generation via a molecular-clutch to drive cells forward (99). Synchronous acto-myosin contractility occurs at the rear of the cell which facilitates the disassembly of trailing adhesion sites whilst also propelling the cell body and nucleus forward to drive migration (50, 100).

An important facet of mesenchymal migration is the focalization of matrix degradation, which is dependent on targeted matrix metalloprotease (MMP) secretion. A landmark study by Wolf and Friedl, identified MMP focalization to the neck of pseudopodia (101). Although the mechanism of MMP targeting during collagenolysis is still unclear, we have demonstrated that microtubules and the associated +TIP proteins, CLASPs, play a crucial role in the release of ECM-matrix interactions via targeted delivery of MMPs to adhesion sites (Figure 1) (102). We have recently identified that CLASPs are overexpressed in several metastatic melanoma cell lines, supported by gene expression database analysis (103–105), outlining a potential role for this process to be dysregulated in metastatic melanoma. Understanding the mechanisms governing MMP-targeted secretion in the context of 3D models will elucidate how cells co-ordinate polarized proteolysis to drive invasion in response to the microenvironment. Nuclear-deformation is also thought to play a mechanosensitive rate limiting role as nuclear deformation has been shown to arrest migration if proteolysis is inhibited (75, 76). Although microtubules are less well understood in the context of 3D migration, we know that confined migration requires microtubules (106) and microtubules are mechanically coupled to the nuclear envelope (107). This in addition to microtubule roles in trafficking, polarity and spatio-temporal regulation of signaling, highlight that microtubules are likely to play key roles in melanoma invasion (60, 63, 108).

## The role of mechanoreciprocity in migration mode switching

Binary concepts like Epithelial to Mesenchymal Transition (EMT), and the reverse (MET), have been extensively studied in the fields of development and cancer (109). Our current understanding of cell motility in complex environments highlights that the initiation of invasion is not as simple as a transcriptional program switch and is heavily influenced by mechanochemical signaling and the plasticity it evokes. Until recently, the two dichotomous modes of 3D migration, mesenchymal or amoeboid, were thought to be independent. We now know they are likely to be a continuum of a single migration process whereby cells dynamically switch between modes (Figure 1). This adaptive response is highlighted in cases whereby cells are subjected to continuous vs. non-continuous confinement, which catalyze very different migration phenotypes (Figures 2A,B). Continuous confinement is analogous to the cellular movement in a parallel, tube-like, structure or between two planes of tissue, resulting in cellular preference for nuclear piston-driven lobopodial migration (110, 111); whereas, non-continuous confinement is analogous to cells traversing between networks of fibers resulting in squeezing-based amoeboid migration (Figures 2Bi,ii) (50). Importantly, the premise of mechanoreciprocity and migrational plasticity has the potential to encompass many more modes of migration that we do not yet fully understand and have not yet discovered.

## Melanoma-stroma interaction

Melanoma cell invasion is facilitated by dynamic interactions between melanoma, ECM (112) and stroma (113, 114), to which the mechanics of ECM play key roles (115). We now know the surrounding tumor stroma is considered to be equally important as the tumor itself (116, 117). This understanding encompasses findings that demonstrate that during disease progression, tumor cells do not act in isolation, instead reciprocally interacting with the surrounding stroma to facilitate tumor cell dissemination. This process occurs through tumor recruitment of stromal cells (117–119), causing stromal cells to co-evolve with the primary tumor which triggers the metastatic cascade (116). This tumor-stroma cross talk involves a “melting pot” of extracellular matrices, fibroblasts, adipocytes, pericytes, endothelial, immune, and inflammatatory cells, such as macrophages and neutrophils (117, 120). The process of stromal cell recruitment is facilitated by tumor-secreted growth factors in a continual paracrine fashion (52). This disruption of the finely-tuned balance of stromal homeostasis and elicits transformed stromal cells to alter the tumor microenvironment to facilitate permissive conditions for tumor cell invasion (116). In a recent study, transformed cancer-associated fibroblasts (CAFs), were shown to facilitate tumor invasion through integrin αVβ3-dependent fibronectin secretion, which induced mechanical changes in the ECM through the contraction of collagen fibers (Figure 1) (105). The geometrical and mechanical ECM changes have been shown to affect the initial onset of invasion at the tumor-ECM interface (108, 109). Reciprocally, stromal secretion of growth factors elicits changes in primary tumor cell behavior, resulting in tumor cell secretion of proteolytic enzymes, including MMPs (116, 121) which have both catalytic and non-catalytic functions in melanoma invasion (32). Tumor-stromal crosstalk also plays a role in resistance to therapies where CAF-dependent ECM remodeling provides a therapeutic “safe-haven” for BRAF mutant melanoma from the BRAF inhibitor PLX4720 (122). This protective stromal signaling was mediated by reactivation of ERK and MAPK, via adhesion-dependent β1-integrin-FAK-Src signaling.

In summary, the many targets that drive metastatic melanoma motility are complex, diverse and outline biological heterogeneity. Our review highlights insight into the idea that motility and survival are not mechanistically separate and thus identifying targets which prevent melanoma migration, may also act to interrupt pro-survival pathways, strengthening the idea behind “mechanomedicines” for cancer (53). The search for migrastatics aims to synthesize the many findings that drive aberrant motility in melanoma to identify a crucial mechanism that melanoma cells engage that is not only specifically dysregulated, but the inhibition of such pathway is inescapable for halting melanoma motility.

## Author contributions

RJ wrote this mini review under the guidance of SS and NH.

### Conflict of interest statement

The authors declare that the research was conducted in the absence of any commercial or financial relationships that could be construed as a potential conflict of interest.
